# Knockdown of NHP2 inhibits hepatitis B virus X protein-induced hepatocarcinogenesis via repressing TERT expression and disrupting the stability of telomerase complex

**DOI:** 10.18632/aging.103810

**Published:** 2020-10-12

**Authors:** Shuming Tang, Weigang Wu, Haoqiang Wan, Xuecheng Wu, Haixia Chen

**Affiliations:** 1 Department of Clinical Laboratory, Shenzhen People’s Hospital, The Second Clinical Medical College, Jinan University, The First Affiliated Hospital, Southern University of Science and Technology, Shenzhen 518020, Guangdong, P.R. China; 2 Department of Infectious Disease, Shenzhen People’s Hospital, The Second Clinical Medical College, Jinan University, The First Affiliated Hospital, Southern University of Science and Technology, Shenzhen 518020, Guangdong, P.R. China; 3 Department of Pathology, Shenzhen People’s Hospital, The Second Clinical Medical College, Jinan University, The First Affiliated Hospital, Southern University of Science and Technology, Shenzhen 518020, Guangdong, P.R. China

**Keywords:** hepatitis B virus X protein, hepatocellular carcinomas, telomerase, telomerase reverse transcriptase, NHP2

## Abstract

Hepatitis B virus X protein (HBx) is highly expressed in HBV-infected hepatocellular carcinoma (HCC) and upregulates transcriptional activation of telomerase reverse transcriptase (TERT). NHP2 is a component of the telomerase complex and also increased in HCC. However, whether NHP2 could accelerate HCC caused by HBx overexpression remains unknown. This study intended to investigate the effects of NHP2 knockdown on HBx-overexpressed HCC and uncover the potential mechanism. Results showed that after HBx overexpression, the expression of TERT and NHP2 was increased. NHP2 knockdown inhibited cell proliferation, colony formation and telomerase activity, while promoting cell apoptosis in PLC/PRF5 cells with or without HBx overexpression. Moreover, the protein expression of TERT and HBx was inhibited, pro-apoptotic proteins Bax and cleaved-caspase3 expression was enhanced, whereas anti-apoptotic protein Bcl-2 level was reduced upon NHP2 silencing in PLC/PRF5 cells with or without HBx upregulation. The interaction between NHP2 and TERT was also confirmed. Treatment with shRNA-NHP2-1 inhibited tumor growth in xenograft model, and the alterations of related proteins were consisted with in vitro results. In conclusion, knockdown of NHP2 could inhibit the proliferation of hepatoma cells overexpressing HBx via inhibiting TERT expression.

## INTRODUCTION

Hepatocellular carcinoma (HCC) is a malignant tumor with high incidence globally. There are over 600,000 new cases diagnosed as HCC every year [1]. It is the fifth most common tumor and the second leading cause of cancer deaths in the world [2]. The major risk factors for HCC include hepatitis B virus (HBV) or hepatitis C virus (HCV) infection, cirrhosis, alcohol, aflatoxin-contaminated food, and certain genetic diseases such as hemochromatosis and various metabolic abnormalities [3]. Although safe and effective HBV vaccines are available, persistent HBV infection remains one of the most important pathogenic agents, especially in Asia and Africa [4]. Previous epidemiologic studies revealed that the HCC incidence for HBV carriers is 25-37 times than that for uninfected people, and HBV infection also contributes to the invasion, metastasis and development of HCC [5]. These findings indicated an etiological connection of persistent HBV infection with HCC.

With the development of biotechnology and increasing numbers of studies about HBV-associated HCC, the causal relationship between HBV infection and HCC has been firmly established and the molecular mechanisms of HBV-induced HCC have been widely clarified. On the one hand, the integration of HBV DNA into the host genome can cause genomic instability, thereby initiating hepatocarcinogenesis [6]. On the other hand, the interaction between HBV and immune cells may result in persistently existing and amplified hepatic inflammatory response, which plays a considerable role in tumor occurrence or development [6, 7]. Hepatitis B virus X, encoded by the HBV X gene, is termed HBx. HBx, required for the virus infection, has been believed to function in the pathogenesis of HCC [8]. As studies have shown, HBx expression may boost hepatocarcinogenesis by reducing telomerase activity during hepatoma cell proliferation, which is supported by breakpoint analysis of the HBV genome [7]. HBx protein may up-regulate the transcriptional activation of human telomerase transcriptase (TERT), which is possibly the mechanism in HCC [9, 10]. Besides, HBx can serve as a transcriptional activator or suppressor and prevent or promote hepatocyte apoptosis [11, 12]. However, the exact molecular mechanisms of HBx-induced HCC are incompletely understood.

TERT is located on chromosome 5p. HBV DNA is integrated near TERT, which activates expression of the TERT gene to promote the conversion of tumor cells and provoke HCC [13]. Data from whole-genome sequencing have confirmed the frequent observation of HBV genome integration in the TERT locus in a high clonal proportion [14, 15]. Human TERT is a catalytic subunit of telomerase and uses the RNA component hTR as a substrate to generate telomere sequences. It is closely related to immortalization of cells, tumorigenesis and senescence of cells. The TERT protein forms a complex with other essential factors, the telomerase RNA component (TERC) and accessory proteins, such as dyskerin (DKC1), TCAB1, NHP2, NOP10 and GAR1, the deletion of any of which will generate instability of the telomerase complex and inhibition of telomerase activity [16, 17]. It is noteworthy that, in GEPI online database, NHP2 displays higher expression in tissues of HCC compared with normal hepatic tissues, indicating the potential regulatory effect of NHP2 on HCC.

In the present study, we knocked down NHP2 expression in PLC/PRF5 hepatoma cell lines with or without HBx overexpression to investigate the regulatory relationship between NHP2 and HBx-induced HCC as well as explore the potential mechanisms. At the same time, the above PLC/PRF5 hepatoma cell lines were transplanted into nude mice to construct xenograft model, followed by injection of shRNA-NHP2, to observe the actions of NHP2 *in vivo*.

## RESULTS

### Stable overexpression of HBx in PLC/PRF5 hepatoma cells increases the level of TERT and NHP2

**Figure 1:**
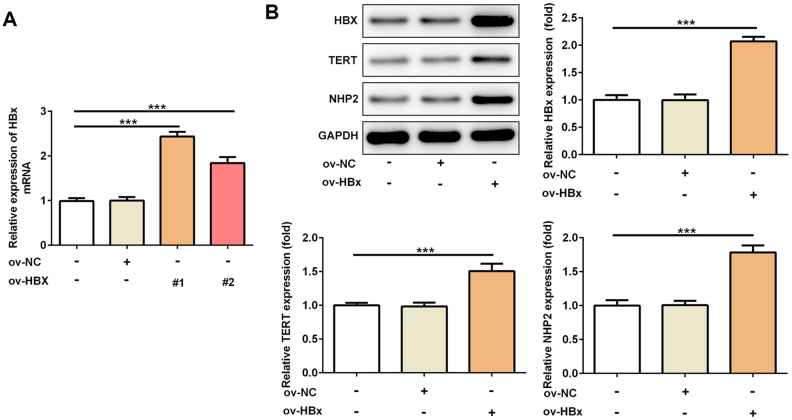
**Overexpression of HBx in PLC/PRF5 hepatoma cells increases TERT and NHP2 expression.** (**A**) The HBx mRNA expression was determined after PLC/PRF5 cells were transfected with ov-NC, ov-HBx-1 or ov-HBx-2 plasmids. (**B**) The protein expression of HBx, TERT and NHP2 was measured in the absence or presence of HBx stable expression. Ov: overexpression, NC: negative control, HBx: Hepatitis B virus X, TERT: telomerase reverse transcriptase. ^***^P<0.001.

To confirm whether HBx could directly affect TERT and NHP2 expression, we constructed two vectors to overexpress HBx in PLC/PRF5 hepatoma cells. Result from Figure 1A verified the successful overexpression of HBx. OverExp-HBx-1 was chosen for establishing stable HBx-overexpressing PLC/PRF5 cells based on its higher efficacy. As shown in Figure 1B, the protein expression of TERT and NHP2 was higher in PLC/PRF5 cells that stably expressing HBx compared with normal and negative control (NC) PLC/PRF5 cells. These results confirmed the promotive effect of HBx on TERT and suggested the active role of NHP2 in HBx-induced HCC.

### Knockdown of NHP2 inhibits proliferation while promotes apoptosis in PLC/PRF5 hepatoma cells with or without HBx overexpression

**Figure 2:**
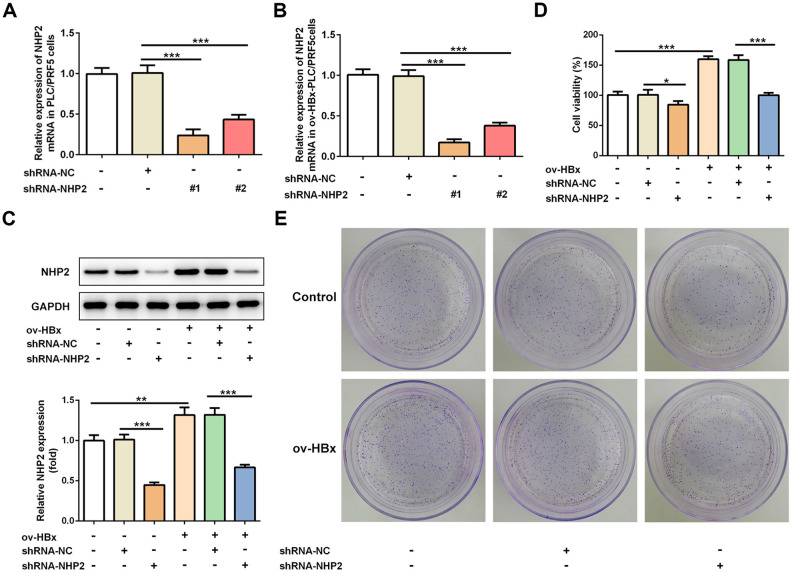
**Knockdown of NHP2 inhibits proliferation of PLC/PRF5 hepatoma cells with stable HBx overexpression or not.** (**A**) The NHP2 mRNA was determined after PLC/PRF5 cells were transfected with shRNA-NC, shRNA-NHP2-1 or shRNA-NHP2-2. (**B**) The NHP2 mRNA was determined after HBx-overexpressed PLC/PRF5 cells were transfected with shRNA-NC, shRNA-NHP2-1 or shRNA-NHP2-2. (**C**) Representative western blot bands together with quantitative analysis for NHP2 expression in PLC/PRF5 cells of different groups. (**D**) Cell viability of PLC/PRF5 cells that stably overexpressed HBx or not in the absence or presence of NHP2 knockdown. (**E**) Representative colony formation assay of PLC/PRF5 cells from various groups. Ov: overexpression, NC: negative control, HBx: Hepatitis B virus X. ^*^P<0.05, ^**^P<0.01 and ^***^P<0.001.

To clarify the effects of NHP2 on HBx-induced HCC, two pairs of chemically synthesized shRNAs (shRNA1 and shRNA2) targeting NHP2 and negative control (sh-NC group) were transfected into PLC/PRF5 hepatoma cells subjected to OverExp-HBx-1 transfection or not, respectively. The results of RT-qPCR showed that compared with the blank control, the expression of NHP2 was obviously inhibited by both shRNAs. The inhibition efficiency of shRNA-1 was higher than that of shRNA-2 (Figure 2A and 2B). Therefore, shRNA-NHP2-1 was chosen for subsequent experiments, and results from western blot further confirmed the knockdown of NHP2 protein (Figure 2C).

**Figure 3:**
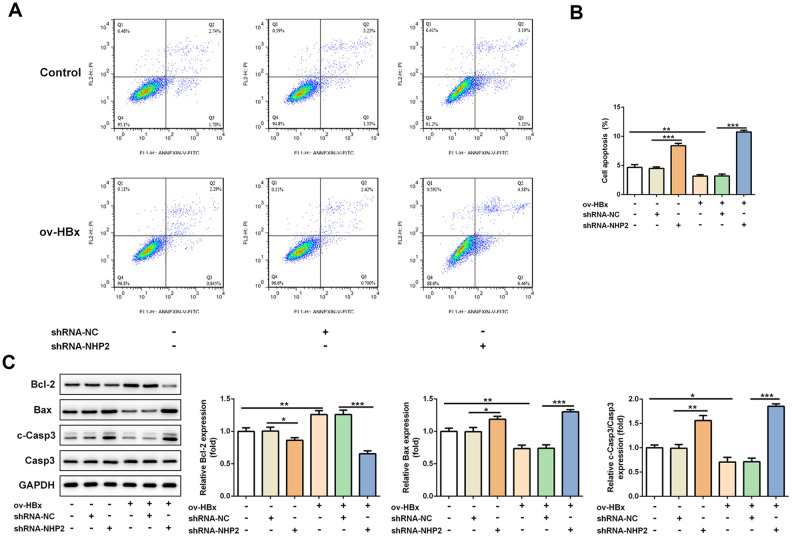
**Knockdown of NHP2 promotes apoptosis of PLC/PRF5 hepatoma cells stably overexpressing HBx or not.** (**A**) Representative flow cytometry assay of PLC/PRF5 cells with or without HBx promotion in the absence or presence of NHP2 silencing. (**B**) Quantitative analysis for cell apoptosis from flow cytometry assay. (**C**) Representative western blot bands together with quantitative analysis for Bcl-2, Bax and cleaved-Caspase 3 expression of PLC/PRF5 cells in the aforementioned groups. Ov: overexpression, NC: negative control, HBX: Hepatitis B virus X. ^*^P<0.05, ^**^P<0.01 and ^***^P<0.001.

The effect of NHP2 on the proliferation of PLC/PRF5 cells that stably overexpressed HBx or not was detected by CCK-8 and colony formation assays. Compared with the blank and NC groups, the cell viability was significantly reduced after NHP2 knockdown regardless whether HBx were overexpressed in PLC/PRF5 cells (Figure 2D). Consistent with the results of the CCK-8 assay, the number of clones formed in shRNA-NHP2-1-transfected cells was markedly decreased in comparison with the blank and NC groups (Figure 2E). Moreover, flow cytometry revealed that knockdown of NHP2 increased the proportion of apoptosis in PLC/PRF5 cells under HBx promotion or not (Figure 3A and 3B). Further, the expression of apoptosis-associated proteins including Bax, Bcl-2 and cleaved-caspase3 was detected. The data showed that the protein expression levels of Bax and cleaved-caspase3 were significantly enhanced, while the expression of anti-apoptotic protein Bcl-2 was obviously down-regulated in the NHP2-silenced cells relative to those in blank or NC groups (Figure 3C), indicating the enhancement of NHP2 silence on apoptosis in PLC/PRF5 cells in the absence or presence of HBx elevation.

### Knockdown of NHP2 reduces telomerase activity in PLC/PRF5 hepatoma cells that stably overexpressed HBx or not via interfering with TERT expression

**Figure 4:**
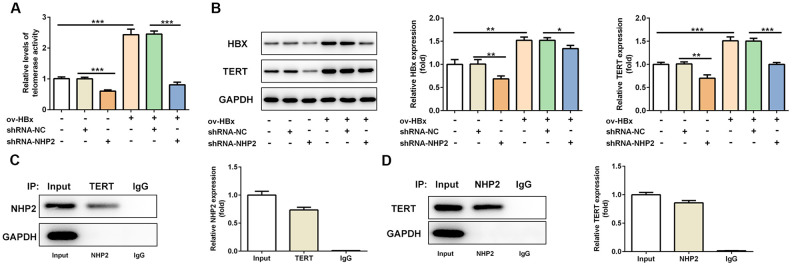
**Knockdown of NHP2 reduces telomerase activity of PLC/PRF5 cells with or without HBx upregulation via interfering with TERT expression.** (**A**) The telomerase activity of PLC/PRF5 cells with or without HBx upregulation in the absence or presence of NHP2 knockdown. (**B**) Representative western blot bands together with quantitative analysis for HBx and TERT expression in different groups of PLC/PRF5 cells. (**C**, **D**) The interaction between TERT and NHP2 was determined by co-immunoprecipitation assay. Ov: overexpression, NC: negative control, HBX: Hepatitis B virus X. ^*^P<0.05, ^**^P<0.01 and ^***^P<0.001.

Next, we explored the alterations of telomerase activity under the circumstance of NHP2 knockdown. We found reduced telomerase activity in shRNA-NHP2-1 PLC/PRF5 cells compared with control or shRNA-NC groups (Figure 4A and 4B). In addition, we detected the expression changes of TERT and HBx protein, and discovered that both TERT and HBx expression was cut down in the presence of shRNA-NHP2-1 (Figure 4C). These results illustrated that knockdown of NHP2 could disturb the activity of telomerase. At the same time, the direct interaction between NHP2 and TERT was confirmed by Co-IP assay (Figure 4D and 4E).

### Knockdown of NHP2 suppresses tumor growth and promotes apoptosis in xenograft model undergoing injection with or without HBx-overexpressed PLC/PRF5 hepatoma cells

**Figure 5:**
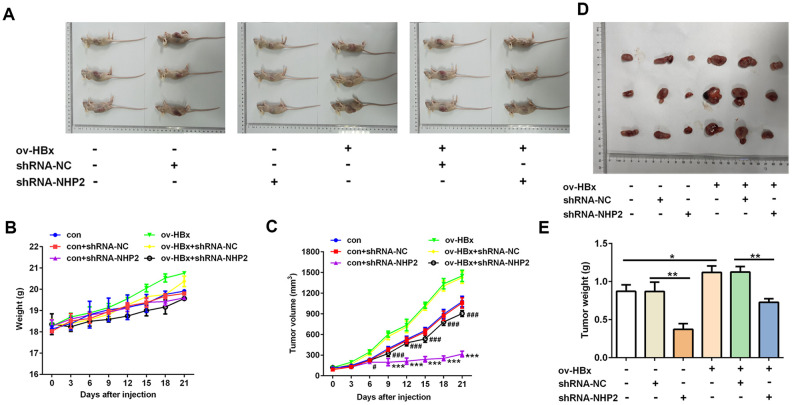
**Knockdown of NHP2 suppresses tumor growth in xenograft model injected with or without HBx-overexpressed PLC/PRF5 hepatoma cells.** (**A**–**C**) After PLC/PRF5 cell lines with or without HBx upregulation were injected into the nude mice, the body weight (**B**) and the tumor volume (**C**) were measured every three day and plotted as the mean ± SD (*n* = 7); ^***^ P < 0.001 vs con + shRNA-NC, ^#^P<0.05 and ^###^P < 0.001 vs ov-HBx + shRNA-NC. (**D**, **E**) At the 21 day after first injection, the tumors were isolated, the tumor weight was weighed. ^*^P<0.05 and ^**^P<0.01.

Finally, in order to verify our findings in vivo, we injected PLC/PRF5 cells stably overexpressing HBx or not into nude mice to generate xenograft model. Then, shRNA-NHP2-1 or scramble shRNAs were injected into tumor tissues of mice. As shown in Figure 5, the tumor volume and tumor weight of mice that were treated with shRNA-NHP2-1 was significantly smaller than those of mice treated with scramble shRNA or normal saline (control), no matter whether HBx-overexpressing PLC/PRF5 cells were injected. These results demonstrated that knockdown of NHP2 could suppress the tumor growth of HCC regardless of HBx upregulation or not.

**Figure 6:**
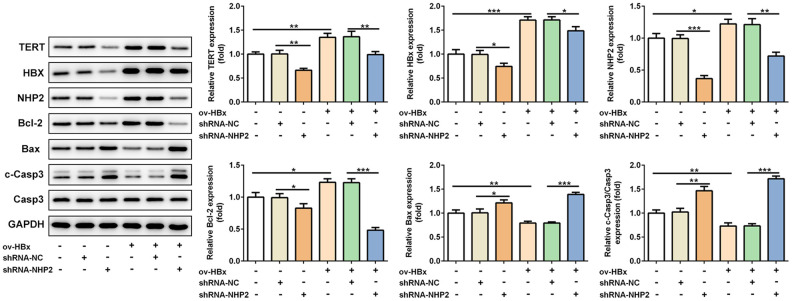
**Knockdown of NHP2 promotes tumor tissues apoptosis in xenograft model that were injected with or without HBx-overexpressed PLC/PRF5 hepatoma cells.** Representative western blot bands together with quantitative analysis for TERT, HBx, NHP2, Bcl-2, Bax and cleaved-Caspase 3 expression in different types of tumor tissues. Ov: overexpression, NC: negative control, HBX: Hepatitis B virus X. ^*^P<0.05, ^**^P<0.01 and ^***^P<0.001.

Besides, the expression levels of related proteins in tumor tissues were also evaluated. Results from Figure 6 revealed that, the expression of TERT, in mice tissues with HBx enhancement or not, HBx as well as NHP2 was blunted, Bax and cleaved-caspase3 levels were enhanced, whereas Bcl-2 expression was reduced after NHP2 inhibition.

## DISCUSSION

HCC is the leading cause of cancer-induced mortality all over the world, however, the crucial mechanism underlying HCC is largely unknown. HBV infection is closely related to occurrence and development of HCC, and HBx functions as a multifunctional regulatory protein associated with hepatocellular carcinogenesis [18]. Although it does not directly bind to DNA, HBx enhances the activity of nuclear transcription factors via interaction with specific proteins [19, 20]. HBx can interact with p53 to inhibit its normal function and expression of its target genes associated with apoptosis [20]. In addition, HBx can activate multiple oncogenic signaling pathways, including the NF-κB, JAK/STAT, Ras/Raf/MAPK (mitogen-activated protein kinase), PI3K (phosphatidylinositol 3 kinase)/Akt, and Wnt/β-catenin pathways [21, 22]. Notably, data from several studies reveled that HBx also enhances telomerase activity, although its effects are under debate [23]. Compelling evidence has suggested that HBx activates telomerase activity via up-regulating TERT mRNA expression. HBx is also proved to induce telomere shortening by acting as a transcriptional corepressor of myc-associated zinc finger (MAZ) protein on the TERT promoter [24]. In the present study, we constructed HBx-overexpressing PLC/PRF5 cells, which disclosed that the expression of TERT and NHP2 together with telomerase activity was highly promoted, and the proliferation ability was enhanced, whereas, the apoptotic level was inhibited. Furthermore, the tumor volume of nude mice with injection of HBx-overexpressing PLC/PRF5 cells was larger than that of mice with injection of PLC/PRF5 cells without HBx overexpression. These results were in accordance with previous studies, in which HBx was suggested to function as a promoter in hepatocellular carcinogenesis and enhance the activity of telomerase and TERT via trans-activation [9, 20, 25, 26].

Although a study from Su et al unexpectedly found the HBx-mediated suppression on human telomerase promoter, their findings have also provided evidence for the oncogenic property of HBx [27]. In a word, the active effects of HBx on telomerase and TERT have been fully evidenced by others’ and our studies. Therefore, to disturb the actions of HBx on TERT may be a valuable therapy for treating HBV-induced HCC. NHP2 is the component of TERT complex, in which the deficiency of any component will lead to its instability [28]. Importantly, the increased expression of NHP2 is related to hepatocarcinogenesis. Herein, we speculated that knockdown of NHP2 would inhibit HBx-induced hepatocarcinogenesis via decreasing TERT expression and disrupting the stability of telomerase complex. HBx was promoted in human hepatoma cell line PLC/PRF5 to simulate HBx-induced HCC, and shRNA-NHP2-1 was subsequently transfected into cells to knockdown NHP2. As expected, our results unveiled that the proliferation ability together with telomerase activity and TERT expression were significantly reduced, while apoptotic level was obviously enhanced in the presence of NHP2 knockdown. Results from in vivo xenograft model also confirmed the findings of in vitro. These results displayed that knockdown of NHP2 could actually prevent hepatocarcinogenesis induced by HBx overexpression. In addition, we also confirmed the interaction between NHP2 and TERT, indicating the modulatory effect of NHP2 knockdown on TERT expression. Interestingly, we also observed that in PLC/PRF5 cells without HBx overexpression, knockdown of NHP2 could still exert anti-hepatocarcinogenic effects both in vitro and in vivo. The protective role of NHP2 repression in PLC/PRF5 cells without HBx overexpression was lower than that in HBx-overexpressing PLC/PRF5 cells. These results implied that knockdown of NHP2 could prevent the hepatocarcinogenesis despite of HBx expression pattern, which could be easily explained considering that the reactivation of TERT can promote the conversion of tumor cells and cause HCC [13]. However, the specific mechanisms involved in the actions of NHP2 on TERT and telomerase activity remain to be clarified. Moreover, in this study, all the experiments were performed on a single cell line PLC/PRF5, further *in vitro* and *in vivo* studies need to be carried out using other hepatoma cell lines.

Taken together, in the present study, we for the first time revealed the modulatory effect of NHP2 on HBx-induced HCC. Our results showed that knockdown of NHP2 could suppress hepatocarcinogenesis associated with HBx overexpression both *in vitro* and *in vivo*, which could be explained by the disruption of TERT complex stability. Therefore, inhibition of NHP2 expression may provide a novel therapeutic approach for treating or preventing HBx-induced HCC.

## MATERIALS AND METHODS

### Cell culture

Human hepatoma cell line PLC/PRF5 was incubated with DMEM medium supplemented with 10% FBS and 1% antibiotics (penicillin and streptomycin) in an environment of 37°C containing 5% CO_2_. The medium was changed within 1 to 3 days depending on the growth of the cells, and cells were passaged under cell confluence more than 85%.

### Cell transfection

The full length HBx sequence was constructed in mammalian expression vector pcDNA3.1 (Invitrogen, USA) and then transfected into PLC/PRF5 cells using Lipofectamine 2000 reagents (Invitrogen, USA) according to manufacturer’s protocols. At 48 h after transfection, the transfected cells were grown in selection medium containing 500 mg/ml G418. PLC/PRF5 cells stably overexpressing HBx were selected after formation of resistant clones. Cells that transfected with empty pcDNA3.1 vectors were used as negative control.

NHP2 short hairpin (sh)-RNA plasmids (CCGGGTCATGTGTGAGGACCGAAATC-TCGAGATTTCGGTCCTCACACATGACTTTTTG; Santa Cruz Biotechnology, USA) or scrambled shRNA (shRNA-NC) were transfected into PLC/PRF5 cells by Lipofectamine 2000 reagent (Invitrogen, USA). The NHP2 expression was assessed by real-time quantitative polymerase chain reaction (RT-qPCR) assays.

### CCK-8

For the assessment of cell proliferation, PLC/PRF5 cells stably transfected with HBx or empty vectors in the absence or presence of NHP2 knockdown were analyzed by Cell Counting Kit-8 (CCK-8). At 48 h post-transfection, 10μl CCK-8 working solution was added to each well, followed by incubation for 2 h under normal cell culture condition. Finally, the optical density (OD) was evaluated at the 450 nm wavelength using a microplate reader.

### Colony formation

For the assessment of colony formation, cell suspension was resuspended with 1 ml DMEM medium in the presence of 20% FBS and 0.9% methylcellulose medium (Sigma, USA). After being incubated in 24-well plates for 2 weeks, a colony with more than 50 cells was counted as a positive colony. The colony forming ability was calculated by a percentage of the number of colonies in the test group to the control group.

### Western blot

Protein extraction from cultured PLC/PRF5 cells was performed using RIPA lysis buffer (Thermo Fisher Scientific, Inc.). Total proteins from tumor tissues were extracted using lysis buffer containing 10 mM Tris PH 7.6, 50 mM NaCl, 1 mM EDTA, 1 mM phenylmethanesulfonyl fluoride (PMSF), 1% TritonX-100, 2.5 mM sodium pyrophosphate, 1% Na_3_VO_4_, 0.5 μg/ml leupeptin and other phosphatase inhibitors, and homogenized by an electric homogenizer.

All protein samples were quantified by a bicinchoninic acid kit (Thermo Fisher Scientific, Inc) and equal amounts of protein were subjected to SDS-PAGE, followed by transferring onto PVDF membranes (Bio-Rad Laboratories, Inc.). The membranes were blocked with 5% non-fat milk at 37°C for 1 h, after which the membranes were cultivated with the following primary antibodies (Abcam) overnight at 4ΰC: anti-HBx (ab39716, 1:500), anti-TERT (ab32020, 1:1000), anti-NHP2 (ab180498, 1:1000), anti-Bcl2 (ab59348, 1:1,000), anti-Bax (ab32503, 1:1,000), anti-cleaved caspase-3 (ab2302, 1:500) and anti-GAPDH (ab8245, 1:10000). Then, the membranes were incubated with HRP-conjugated goat anti-rabbit IgG (1:10000) for 2 h at room temperature. The results were visualized using electrochemiluminescence system (Amersham Imager 600; GE Healthcare). Image J software (1.8.0_112, National Institutes of Health) was utilized for densitometric analysis of western blot.

The interaction of TERT and NHP2 in PLC/PRF5 cells was validated by co-immunoprecipitation (Co-IP) method. For Co-IP assay, the soluble protein samples were pre-incubated with protein G/A-agarose (Cell signaling Biotechnology, Germany) at 4°C overnight and then incubated with 100μL of protein G/A-agarose pre-coupled to primary antibodies for at least 3 h. The mixtures were then washed, boiled, and subjected to western blot.

### Flow cytometry

Cell apoptosis was assessed by flow cytometry using propidium iodide (PI) staining. Briefly, cells were gently washed twice with PBS, digested with 0.25% trypsin and centrifuged at 200 × g for 5 min. After the resuspension of the cell pellet with 1 ml NaCl/Pi supplemented with 100μg/ml RNase, the cells were incubated with PI for 15 min in the dark and immediately analyzed by flow cytometry (BD FACSCanto П; Becton, Dickinson and Company). Data were dissected by flow cytometry software (FlowJo 7.6; Becton, Dickinson and Company).

### Detection of telomerase activity

The isolation of genomic DNA was finished using DNAzol regent (Cat. NO. 10503-027; Invitrogen; Thermo Fisher Scientific, Inc) according to the manufacture’s instruction. Then, the telomerase activity was detected by RT-qPCR using kits (Cat. NO. KGA1028H; KeyGEN BioTECH). The following thermocycling conditions were used for the qPCR: Initial denaturation at 95ΰC for 10 min; and 40 cycles of 95ΰC for 15 sec and 60ΰC for 30 sec, followed by default of melt curve (Applied Biosystems 7500; Thermo Fisher Scientific, Inc.).

### Nude mice xenograft model

All animal studies were performed in accordance with the Care and Use Guide of Laboratory Animals of the National Institutes of Health, with the approval of the Animal Studies Ethics Committees of the Shenzhen People’s Hospital (IACUC-20180822-1). PLC/PRF5 cell lines stably expressing HBx or not were injected into the six-week old male BALB/C nude mice (Animal Experiment Center of Shenzhen People’s Hospital). When the average tumor diameter reached 0.4-0.5 cm, shRNA-NHP2 or shRNA-NC was injected into tumor every 3 days for three times. Control mice were injected with normal saline. At the 21 day after first injection, all mice were euthanized, tumors were removed, and tumor volume was calculated by the following equation: tumor volume (mm^3^) = length (mm) × width^2^ (mm^2^) /2.

### Statistical analysis

Data were derived from at least three independent experiments and were presented as mean ± standard deviation. The statistical analyses were conducted using GraphPad Prism 6 (GraphPad Software, Inc.) and group statistical comparisons were assessed by one-way ANOVA followed by Turkey’s post hoc test. P<0.05 was considered to indicate statistically significant differences.

## References

[1] Torre LA, Bray F, Siegel RL, Ferlay J, Lortet-Tieulent J, Jemal A. Global cancer statistics, 2012. CA Cancer J Clin. 2015; 65:87–108. 10.3322/caac.21262 25651787

[2] Ferlay J, Soerjomataram I, Dikshit R, Eser S, Mathers C, Rebelo M, Parkin DM, Forman D, Bray F. Cancer incidence and mortality worldwide: sources, methods and major patterns in GLOBOCAN 2012. Int J Cancer. 2015; 136:E359–86. 10.1002/ijc.29210 25220842

[3] Zhu RX, Seto WK, Lai CL, Yuen MF. Epidemiology of hepatocellular carcinoma in the Asia-pacific region. Gut Liver. 2016; 10:332–39. 10.5009/gnl15257 27114433 PMC4849684

[4] Liu CJ, Kao JH. NOhep: toward global control of hepatitis B virus infection-an introduction. J Infect Dis. 2017; 216:S749. 10.1093/infdis/jix313 29156045

[5] Xu C, Zhou W, Wang Y, Qiao L. Hepatitis B virus-induced hepatocellular carcinoma. Cancer Lett. 2014; 345:216–22. 10.1016/j.canlet.2013.08.035 23981576

[6] Levrero M, Zucman-Rossi J. Mechanisms of HBV-induced hepatocellular carcinoma. J Hepatol. 2016; 64:S84–101. 10.1016/j.jhep.2016.02.021 27084040

[7] Wang M, Xi D, Ning Q. Virus-induced hepatocellular carcinoma with special emphasis on HBV. Hepatol Int. 2017; 11:171–80. 10.1007/s12072-016-9779-5 28097530

[8] Liu Y, Feng J, Sun M, Yang G, Yuan H, Wang Y, Bu Y, Zhao M, Zhang S, Zhang X. Long non-coding RNA HULC activates HBV by modulating HBx/STAT3/miR-539/APOBEC3B signaling in HBV-related hepatocellular carcinoma. Cancer Lett. 2019; 454:158–70. 10.1016/j.canlet.2019.04.008 30981758

[9] Liu H, Shi W, Luan F, Xu S, Yang F, Sun W, Liu J, Ma C. Hepatitis B virus X protein upregulates transcriptional activation of human telomerase reverse transcriptase. Virus Genes. 2010; 40:174–82. 10.1007/s11262-009-0441-3 20107884

[10] Lin WH, Yeh SH, Yang WJ, Yeh KH, Fujiwara T, Nii A, Chang SS, Chen PJ. Telomerase-specific oncolytic adenoviral therapy for orthotopic hepatocellular carcinoma in HBx transgenic mice. Int J Cancer. 2013; 132:1451–62. 10.1002/ijc.27770 22886913

[11] Wang X, Huo B, Liu J, Huang X, Zhang S, Feng T. Hepatitis B virus X reduces hepatocyte apoptosis and promotes cell cycle progression through the Akt/mTOR pathway in vivo. Gene. 2019; 691:87–95. 10.1016/j.gene.2018.12.054 30630095

[12] Lin S, Zhang YJ. Interference of apoptosis by hepatitis B virus. Viruses. 2017; 9:230. 10.3390/v9080230 28820498 PMC5580487

[13] Cevik D, Yildiz G, Ozturk M. Common telomerase reverse transcriptase promoter mutations in hepatocellular carcinomas from different geographical locations. World J Gastroenterol. 2015; 21:311–17. 10.3748/wjg.v21.i1.311 25574106 PMC4284350

[14] Fujimoto A, Totoki Y, Abe T, Boroevich KA, Hosoda F, Nguyen HH, Aoki M, Hosono N, Kubo M, Miya F, Arai Y, Takahashi H, Shirakihara T, et al. Whole-genome sequencing of liver cancers identifies etiological influences on mutation patterns and recurrent mutations in chromatin regulators. Nat Genet. 2012; 44:760–64. 10.1038/ng.2291 22634756

[15] Schulze K, Imbeaud S, Letouzé E, Alexandrov LB, Calderaro J, Rebouissou S, Couchy G, Meiller C, Shinde J, Soysouvanh F, Calatayud AL, Pinyol R, Pelletier L, et al. Exome sequencing of hepatocellular carcinomas identifies new mutational signatures and potential therapeutic targets. Nat Genet. 2015; 47:505–11. 10.1038/ng.3252 25822088 PMC4587544

[16] Shay JW. Role of telomeres and telomerase in aging and cancer. Cancer Discov. 2016; 6:584–93. 10.1158/2159-8290.CD-16-0062 27029895 PMC4893918

[17] Shay JW, Wright WE. Telomeres and telomerase: three decades of progress. Nat Rev Genet. 2019; 20:299–309. 10.1038/s41576-019-0099-1 30760854

[18] Chi HC, Chen SL, Lin SL, Tsai CY, Chuang WY, Lin YH, Huang YH, Tsai MM, Yeh CT, Lin KH. Thyroid hormone protects hepatocytes from HBx-induced carcinogenesis by enhancing mitochondrial turnover. Oncogene. 2017; 36:5274–84. 10.1038/onc.2017.136 28504722

[19] Minor MM, Slagle BL. Hepatitis B virus HBx protein interactions with the ubiquitin proteasome system. Viruses. 2014; 6:4683–702. 10.3390/v6114683 25421893 PMC4246244

[20] Shlomai A, de Jong YP, Rice CM. Virus associated Malignancies: the role of viral hepatitis in hepatocellular carcinoma. Semin Cancer Biol. 2014; 26:78–88. 10.1016/j.semcancer.2014.01.004 24457013 PMC4048791

[21] Shen L, Zhang X, Hu D, Feng T, Li H, Lu Y, Huang J. Hepatitis B virus X (HBx) play an anti-apoptosis role in hepatic progenitor cells by activating Wnt/β-catenin pathway. Mol Cell Biochem. 2013; 383:213–22. 10.1007/s11010-013-1769-5 23934090

[22] Rawat S, Clippinger AJ, Bouchard MJ. Modulation of apoptotic signaling by the hepatitis B virus X protein. Viruses. 2012; 4:2945–72. 10.3390/v4112945 23202511 PMC3509679

[23] Kim YJ, Yoo JE, Jeon Y, Chong JU, Choi GH, Song DG, Jung SH, Oh BK, Park YN. Suppression of PROX1-mediated TERT expression in hepatitis B viral hepatocellular carcinoma. Int J Cancer. 2018; 143:3155–68. 10.1002/ijc.31731 29987895

[24] Nault JC, Mallet M, Pilati C, Calderaro J, Bioulac-Sage P, Laurent C, Laurent A, Cherqui D, Balabaud C, Zucman-Rossi J. High frequency of telomerase reverse-transcriptase promoter somatic mutations in hepatocellular carcinoma and preneoplastic lesions. Nat Commun. 2013; 4:2218. 10.1038/ncomms3218 23887712 PMC3731665

[25] Kanda T, Goto T, Hirotsu Y, Moriyama M, Omata M. Molecular mechanisms driving progression of liver cirrhosis towards hepatocellular carcinoma in chronic hepatitis B and C infections: a review. Int J Mol Sci. 2019; 20:1358. 10.3390/ijms20061358 30889843 PMC6470669

[26] Lin CL, Kao JH. Review article: the prevention of hepatitis b-related hepatocellular carcinoma. Aliment Pharmacol Ther. 2018; 48:5–14. 10.1111/apt.14683 29722445

[27] Su JM, Lai XM, Lan KH, Li CP, Chao Y, Yen SH, Chang FY, Lee SD, Lee WP. X protein of hepatitis B virus functions as a transcriptional corepressor on the human telomerase promoter. Hepatology. 2007; 46:402–13. 10.1002/hep.21675 17559154

[28] Benyelles M, O’Donohue MF, Kermasson L, Lainey E, Borie R, Lagresle-Peyrou C, Nunes H, Cazelles C, Fourrage C, Ollivier E, Marcais A, Gamez AS, Morice-Picard F, et al. NHP2 deficiency impairs rRNA biogenesis and causes pulmonary fibrosis and høyeraal-hreidarsson syndrome. Hum Mol Genet. 2020; 29:907–22. 10.1093/hmg/ddaa011 31985013

